# Tensiometer-Based Irrigation Management of Subirrigated Soilless Tomato: Effects of Substrate Matric Potential Control on Crop Performance

**DOI:** 10.3389/fpls.2015.01150

**Published:** 2015-12-23

**Authors:** Francesco F. Montesano, Francesco Serio, Carlo Mininni, Angelo Signore, Angelo Parente, Pietro Santamaria

**Affiliations:** ^1^Institute of Sciences of Food Production, National Research Council of ItalyBari, Italy; ^2^Department of Agricultural and Environmental Science, University of Bari Aldo MoroBari, Italy

**Keywords:** irrigation set-point, water relations, through bench system, WUE

## Abstract

Automatic irrigation scheduling based on real-time measurement of soilless substrate water status has been recognized as a promising approach for efficient greenhouse irrigation management. Identification of proper irrigation set points is crucial for optimal crop performance, both in terms of yield and quality, and optimal use of water resources. The objective of the present study was to determine the effects of irrigation management based on matric potential control on growth, plant–water relations, yield, fruit quality traits, and water-use efficiency of subirrigated (through bench system) soilless tomato. Tensiometers were used for automatic irrigation control. Two cultivars, “Kabiria” (cocktail type) and “Diana” (intermediate type), and substrate water potential set-points (−30 and −60 hPa, for “Diana,” and −30, −60, and −90 hPa for “Kabiria”), were compared. Compared with −30 hPa, water stress (corresponding to a −60 hPa irrigation set-point) reduced water consumption (14%), leaf area (18%), specific leaf area (19%), total yield (10%), and mean fruit weight (13%), irrespective of the cultivars. At −60 hPa, leaf-water status of plants, irrespective of the cultivars, showed an osmotic adjustment corresponding to a 9% average osmotic potential decrease. Total yield, mean fruit weight, plant water, and osmotic potential decreased linearly when −30, −60, and −90 hPa irrigation set-points were used in “Kabiria.” Unmarketable yield in “Diana” increased when water stress was imposed (187 vs. 349 g·plant^−1^, respectively, at −30 and −60 hPa), whereas the opposite effect was observed in “Kabiria,” where marketable yield loss decreased linearly [by 1.05 g·plant^−1^ per unit of substrate water potential (in the tested range from −30 to −90 hPa)]. In the second cluster, total soluble solids of the fruit and dry matter increased irrespective of the cultivars. In the seventh cluster, in “Diana,” only a slight increase was observed from −30 vs. −60 hPa (3.3 and 1.3%, respectively, for TSS and dry matter), whereas in “Kabiria,” the increase was more pronounced (8.7 and 12.0%, respectively, for TSS and dry matter), and further reduction in matric potential from −60 to −90 hPa confirmed the linear increase for both parameters. Both glucose and fructose concentrations increased linearly in “Kabiria” fruits on decreasing the substrate matric potential, whereas in “Diana,” there was no increase. It is feasible to act on matric potential irrigation set-points to control plant response in terms of fruit quality parameters. Precise control of substrate water status may offer the possibility to steer crop response by enhancing different crop-performance components, namely yield and fruit quality, in subirrigated tomato. Small-sized fruit varieties benefit more from controlled water stress in terms of reduced unmarketable yield loss and fruit quality improvements.

## Introduction

Irrigation management directly affects crop performance and can lead to qualitative and quantitative improvements in vegetable production (Dukes et al., 2010). Under-irrigation generally results in reduced crop yield and quality, whereas over-irrigation may lead to increased crop vulnerability to diseases, energy costs for water pumping, water loss, and environmental pollution due to fertilizer runoff (Pardossi et al., 2009). Therefore, irrigation management needs to be efficient also in order to help reduce environmental impact and promote sustainable use of resources (Montesano et al., 2015).

Production of vegetable crops in greenhouses has expanded considerably over recent decades in Mediterranean region (Sonneveld and Voogt, 2009; FAO, 2013). Research efforts and the related introduction of technical innovations initially focused on high-quality, healthy products. However, concern over environmentally sustainable production has risen in the last decade, as industrial greenhouse crops are usually seen as entailing high environmental impact (Torrellas et al., 2012). On the other hand, there is also plenty of evidence that greenhouse vegetable production may decrease the environmental impact compared to field cultivation (Stanghellini, 2014). In this framework, providing the greenhouse sector with tools and skills for efficient irrigation management is a key factor.

Among the different approaches proposed to achieve better crop performance and efficient use of resources (water and fertilizers) in irrigated greenhouse agriculture, closed-cycle soilless cultivation, with recycling of nutrient solution (NS) aiming to minimize pollution (Vox et al., 2010), and smart sensor-based irrigation scheduling are promising and increasingly adopted strategies (van Iersel et al., 2013).

Visual assessment of plants and substrate and the use of timers to automate irrigation, still the most common approach in greenhouses, are generally inefficient (Nemali et al., 2007). Among the different possible approaches for improved irrigation management and automation, monitoring the water status of the soil/substrate through sensors, as well as making objective irrigation decisions based on real-time measurements, seems particularly suitable for greenhouse conditions (Jones, 2007; van Iersel et al., 2013). Sensors for measuring water status in the root zone are a dynamic and constantly developing area of technology for both technical and commercial reasons. Although technological developments in soil/soilless water sensors have generally focused on scientific applications, thereby aiming to study the effects of different soil moisture levels on plant physiological responses, interest in using sensors for practical irrigation management has grown in the last decade (Pardossi et al., 2009; Lichtenberg et al., 2013). Sensing soil/soilless substrate water status is becoming easier and more economically feasible, providing opportunities to integrate sensor networks into existing irrigation systems (van Iersel et al., 2013). The basic approach behind the automation of irrigation based on root-zone moisture measurements is simple: the moisture level in the growing media fluctuates according to evaporation and plant water use; sensors detect this change and automatically activate irrigation when the level reaches a set value predetermined by the operator, resulting in on-demand irrigation (van Iersel, 2015).

Two categories of sensors can be used to monitor soil/substrate water status based on measuring two basic inter-related properties: soil volumetric water content sensors measure how much water is present in the soil in terms of volume, whereas soil matric potential sensors measure how tightly water is bound to soil/substrate particles and, as a consequence, the amount of energy the plants need to exert to extract water from a soil or soilless substrate. The relation between volumetric water content and matric potential is characteristic of a specific porous medium and is described by the moisture release curve. Sensing matric potential has the advantage of providing direct information on whether and to what extent water in the growing medium is available to the plants (van Iersel et al., 2013).

While many technological innovations have been introduced into volumetric moisture sensors in the last decade, with a number of reliable and inexpensive sensors principally based on Frequency Domain Reflectometry (FDR) becoming available on the market, the water-filled tensiometer is still the most common sensor for matric potential measurement and has seen little improvement in comparison with the early models, apart from the use of improved pressure transducers and data logging systems (Whalley et al., 2013). Tensiometers are often preferred to other types of substrate moisture sensors due to their low cost, simplicity of use, high accuracy, and direct measurement of matric potential, and also, they are not influenced by temperature and soil osmotic potential; moreover, the possibility of electronic data acquisition through differential pressure transducers (Thalheimer, 2003; Sarkar et al., 2008a; Montesano et al., 2010) makes the tensiometer suitable for automated fertigation control. However, tensiometers must be operated carefully in order to avoid the formation of air bubbles in the shaft; they must be protected from frost and need regular maintenance, for instance, to refill the water in the tube. The risk of cavitation in very dry conditions is also a drawback. When tensiometers are used in pot cultures, some precautions are necessary in order to ensure good contact between the porous tip and the substrate, in particular in soilless conditions where high porosity is common, and to achieve correct sensor positioning, taking into account root distribution and the placing of nozzle(s) in the event of drip irrigation (Pardossi et al., 2009). Tensiometers specifically designed for soilless substrates are also available on the market, equipped with special ceramic tips, thereby allowing for faster equilibration with the surrounding substrate (van Iersel et al., 2013).

Soilless growing media generally hold easily available water (EAW) in a matric potential range from 0 to −100 hPa, with the majority of freely available water from 0 to −50 hPa (de Boodt and Verdonck, 1972; Argo, 1998). Other indications suggest that a matric potential of −200 to −300 hPa could be considered the lower limit for available water for plants, underlining the important role of hydraulic conductivity decrease in dry conditions in soilless substrates (van Iersel et al., 2013). Therefore, for practical purposes, water availability in the growing media can be sensed and measured on the base of matric potential; this has significant implications for the measurement and control of irrigation in soilless conditions, as there is a narrow range of available water for scheduling irrigation (Lea-Cox and Arguedas-Rodriguez, 2011). Matric potential control through tensiometers has also been used to impose controlled water stress in order to improve the quality response of crops in soilless conditions (Sarkar et al., 2008a).

Closed-cycle Zero Runoff Subirrigation (ZRS) is a practical and cost-effective method for achieving efficient water use in potted plants grown in greenhouses in soilless conditions. The basic principle of subirrigation systems is the same: the bottom of the pots is submerged in water (or NS) for a brief period (generally 5–20 min, according to the container size), which gives the growing medium enough time to absorb water by capillarity. An extensive overview on ZRS systems was recently published by Ferrarezi et al. (2015a), to which the readers are referred for a more in-depth review than space will allow herein.

Tomato is the most important greenhouse crop grown in soilless cultivation systems (Savvas et al., 2013). Several studies have been conducted on growing soilless tomato using subirrigation (Santamaria et al., 2003; Incrocci et al., 2006; Sarkar et al., 2008b; Montesano et al., 2010). Subirrigation systems have been successfully automated based on substrate water content using tensiometers (Rouphael et al., 2006; Montesano et al., 2010) as well as capacitance moisture sensors (Ferrarezi et al., 2015b). However, to the best of our knowledge, an evaluation of the overall performance of subirrigated tomato subjected to matric potential control using different irrigation set points has not been conducted.

Given the above considerations, the objective of the present study was to determine the effects of irrigation management based on matric potential control on growth, water relations, yield, quality, and water-use efficiency (WUE) of subirrigated tomato. We hypothesized that using matric potential control through a tensiometer with different irrigation set points would allow us to steer crop performance, with particular reference to WUE, yield, and fruit quality.

## Materials and methods

### Experiment setup and treatment application

The experiment was carried out in a plastic (polymethacrylate) greenhouse at the Experimental Farm “La Noria” of the Institute of Sciences of Food Production (ISPA-CNR) in Mola di Bari, Southern Italy, during a winter-summer growing cycle (January–July, 2005).

Seedlings of two tomato cultivars [*Solanum lycopersicum* L., “Kabiria,” a cocktail type, with small fruits (40–47 mm), and “Diana,” an intermediate type, with large fruits (67–82 mm) (Schwarz et al., 2014)] were transplanted at the second true-leaf stage into 10 L plastic pots (one plant per pot) with bottom holes, filled with 8 L of a perlite [Agrilit 3, Perlite Italiana, Corsico (MI), Italy]: peat (Brill 3 Special, Brill Substrates, Georgsdorf, Germany) mixture (3:1, v:v).

Physical properties (total pore space—TPS, water-holding capacity—WC, air capacity—AC, available water—AW, easily available water—EAW, water-buffer capacity—WBC, and volumetric water content—VWC—at −10, −50, and −100 hPa) were determined according to the European Standard 13040 method (European Standard 13040, 1999) and are reported in Table 1. In brief, the material was equilibrated in water and then transferred into tubes made with two overlapping polyvinyl chloride rings (100 ± 1 mm diameter and 50 ± 1 mm height each). After filling, the double rings were saturated with water for 48 h and then transferred into a sandbox (Eijkelkamp Agrisearch Equipment, Giesbeck, The Netherlands) at −10 cm pressure head (−10 hPa) for 48 h. Thereafter, the double rings were removed from the sandbox and separated. The lower rings were weighted and dried at 105°C to constant mass. EAW and WBC were determined by increasing the values of suction pressure in the sandbox at −50 and −100 cm (−50 and −100 hPa, respectively).

**Table 1:** **Physical properties of the growing medium used in the experiment (perlite:peat 3:1 v:v)**.

**TPS**	**WC**	**AC**	**AW**	**EAW**	**WBC**	**VWC −10 hPa**	**VWC −50 hPa**	**VWC −100 hPa**
**% OF VOLUME**								
94	52	42	13.4	11.1	2.3	52.0	40.9	38.6

*TPS, total pore space; WC, water-holding capacity; AC, air capacity; AW, available water; EAW, easily-available water; WBC, water-buffer capacity; VWC, volumetric water content*.

The minimum (heating) temperature inside the greenhouse was set to 15°C (day) and 10°C (night), whereas above 20°C, the greenhouse temperature was controlled by natural ventilation through the automatic ridge openings. Pots were placed 0.25 m apart at a density of 3.3 plants·m^−2^ on troughs measuring 6 m long and 26 cm wide with 6 cm high sides. The troughs were 1.30 m apart and were covered with polyethylene film and placed on a 2% slope. A 100-L reservoir tank containing NS was placed at the end of each trough [a layout of the subirrigated trough bench system used in the trial is reported by Santamaria et al. (2003)]. NS was recirculated (closed-cycle management) and never discharged at any time during the growing cycle.

The NS was supplied at the top end of each trough (with a flow rate of ≈2.0 L·min^−1^). Excess NS was collected at the base of the troughs and reused for subsequent irrigation events. Reservoir tanks were refilled with fresh NS every 2 days, blending it with the recirculating NS still present in the container. Fertirrigation occurred when the substrate water potential reached the pre-fixed set points (see below); fertirrigation was automatically switched off when water potential increased to values higher than the set points.

The NS was prepared starting from well water with a pH of 7.6. Final macronutrient concentration of the NS was (Mm): N–NO_3_ (10), K^+^ (7.7), P–H_2_${\mathrm{PO}}_{4}^{-}$ (1.9), Mg^2+^ (1.7), Ca^2+^ (3.7), S–${\text{SO}}_{4}^{2-}$ (3.4) resulting in an electrical conductivity (EC) of 2.0 dS·m^−1^. Micronutrients were supplied according to Johnson et al. (1957). The pH of NS was adjusted to 5.5 when needed using 5 M H_3_PO_4_ or KOH.

During the first 2 weeks of the experiment, the fertirrigation schedule was controlled for all treatments by an operation timer; the timing varied from one to two fertirrigations (10 min each) per day.

The experimental treatments were differentiated 15 days after transplanting (DAT). Different irrigation set points based on substrate matric potential were compared: −30 and −60 hPa for “Diana,” and −30, −60, and −90 hPa for “Kabiria.”

Treatments were arranged in a randomized complete design with three replications. Each trough, containing 24 plants, represented an experimental unit. The first two plants at the upper and lower end of each trough were not taken into account for harvesting, sampling, and measurements. Two external rows served as guards.

Tensiometers (LT1 28 cm, Tensio-Technik, Geisenheim, Germany), placed with the porous cup at 7 cm from the bottom of the container (container eight, 26 cm), were used to measure the substrate water potential and to control irrigation. In each treatment, three tensiometers were installed (one per experimental unit/replication; Montesano et al., 2010). Tensiometers were connected to an electronic TensioSwitch (400C, Tensio-Technik) that controlled the beginning and the end of irrigation based on substrate water potential set point. A minimum duration of 3 min, corresponding to a supply of ~200 mL of NS per pot per fertigation, was set.

Plants were trained vertically and topped at the eighth cluster (105 and 131 DAT, respectively, for “Kabiria” and “Diana”), and periodic binding, lateral-stem and basal-leaf pruning was carried out. Pollination was guaranteed by introducing a hive of bumblebees (*Bombus terrestris* L.) into the greenhouse beginning from the anthesis of the first cluster.

Harvests started on 100 DAT and ended on 148 and 156 DAT for “Kabiria” and “Diana,” respectively. For “Kabiria,” each harvest was carried out by picking the entire fruit cluster of a specific order when it had 80% red-ripened fruits on at least 80% of the plants at the experimental unit; for “Diana,” single fruits were harvested at the “breaker” color stage.

### Measurements

#### EC of the substrate aqueous extract

At the end of the cycle, analysis of the growing media was conducted. Substrate was removed from the pot (one pot per experimental unit/replication) and the profile (18 cm from the bottom to the top) was divided horizontally into three equal layers (bottom, middle, and top). Approximately 100 mL of growing medium was sampled from the middle of each layer and an aqueous solution was extracted from the sample, using the 1:1.5 dilution method (Sonneveld et al., 1974). Substrate extract EC was measured using a handheld conductivity meter.

#### Growth analysis

In order to assess the effects of treatments on plant growth, one plant from each experimental unit was sampled on 96 DAT. Plant height, total leaf area (Li-3100, Licor, NE, USA), total fresh and dry weight of leaves, stems, and fruits were determined. Plant material was dried to a constant weight in a forced draft oven at 65°C to determine dry weight. Specific Leaf Area (SLA) was calculated as the ratio between plant leaf area and total plant dry weight.

#### Yield and fruit quality analysis

All harvested fruits were classified into marketable and unmarketable classes (i.e., those showing blossom-end rot or radial cracks on the epicarp). Representative samples of fruits harvested from the second and seventh cluster (105 and 140 DAT for “Kabiria” and 111 and 142 DAT for “Diana,” respectively) were used to determine: (i) fruit total soluble solids (TSS), measured using a portable reflectometer (Brix-Stix BX 100 Hs; TechniQuip Corporation, Livermore, CA, USA) and expressed in °Brix at 20°C; (ii) fruit dry matter percentage (see above); (iii) glucose and fructose (only on the second cluster) concentrations were determined on fruits homogenized at 3000 rpm by ionic chromatography (Dionex model DX500; Dionex Corp., Sunnyvale, CA) with an amperometric detector using a Dionex CarboPac PA1 and isocratic elution with 50 mmol·L^−1^ NaOH (Caretto et al., 2008); K^+^, Mg^2+^, and Ca^2+^ concentrations (on fruits homogenized at 3000 rpm) were determined by ionic chromatography with a conductivity detector, using an IonPack CG12A pre-column and IonPack CS12A separation column (Di Gioia et al., 2013).

#### Plant–water relations

On 92 DAT, water (Ψ_i_), osmotic (Ψ_o_), and turgor (Ψ_t_) *pre-dawn* potentials were measured; on 104 DAT, noon water potential on covered leaf (Ψ_x_) was measured. For all parameters, measurements were replicated three times. Ψ_i_ and Ψ_x_ were measured using a Scholander–Hammel type pressure chamber (Scholander et al., 1965). Ψ_o_ was measured using a Roebling digital micro-osmometer (Gucci et al., 1991). Ψ_t_ was calculated as the difference between Ψ_i_ and Ψ_o_.

#### Water-use efficiency

NS consumption was volumetrically measured by flow meters and recorded every 2 days, when the reservoir tanks were refilled. The WUE was calculated as the transformation efficiency of water through the cultivation system into yield, as the ratio of total fresh weight of fruits to the total volume of NS consumed by the system (Valenzano et al., 2008; De Pascale et al., 2011).

### Statistical analysis

All data were submitted to analysis of variance using the General Linear Model (GLM Proc; SAS 9.1; SAS Institute, Cary, NC, USA). For this purpose, all experimental factors were considered to be fixed.

According to the research objectives, means were compared using orthogonal contrasts with one degree of freedom (Steel and Torrie, 1988). Two polynomial contrasts were performed between the three levels of substrate water potential set points applied to “Kabiria” (linear and quadratic trend); three more contrasts were performed, depending on which variety or substrate water potential was studied: (1) “Diana” vs. “Kabiria”—on average, for −30 and −60 hPa; (2) −30 vs. −60 hPa; (3) the interaction (“Diana” vs. “Kabiria”) × (−30 vs. −60 hPa).

## Results

### Substrate electrical conductivity

**Figure 1:**
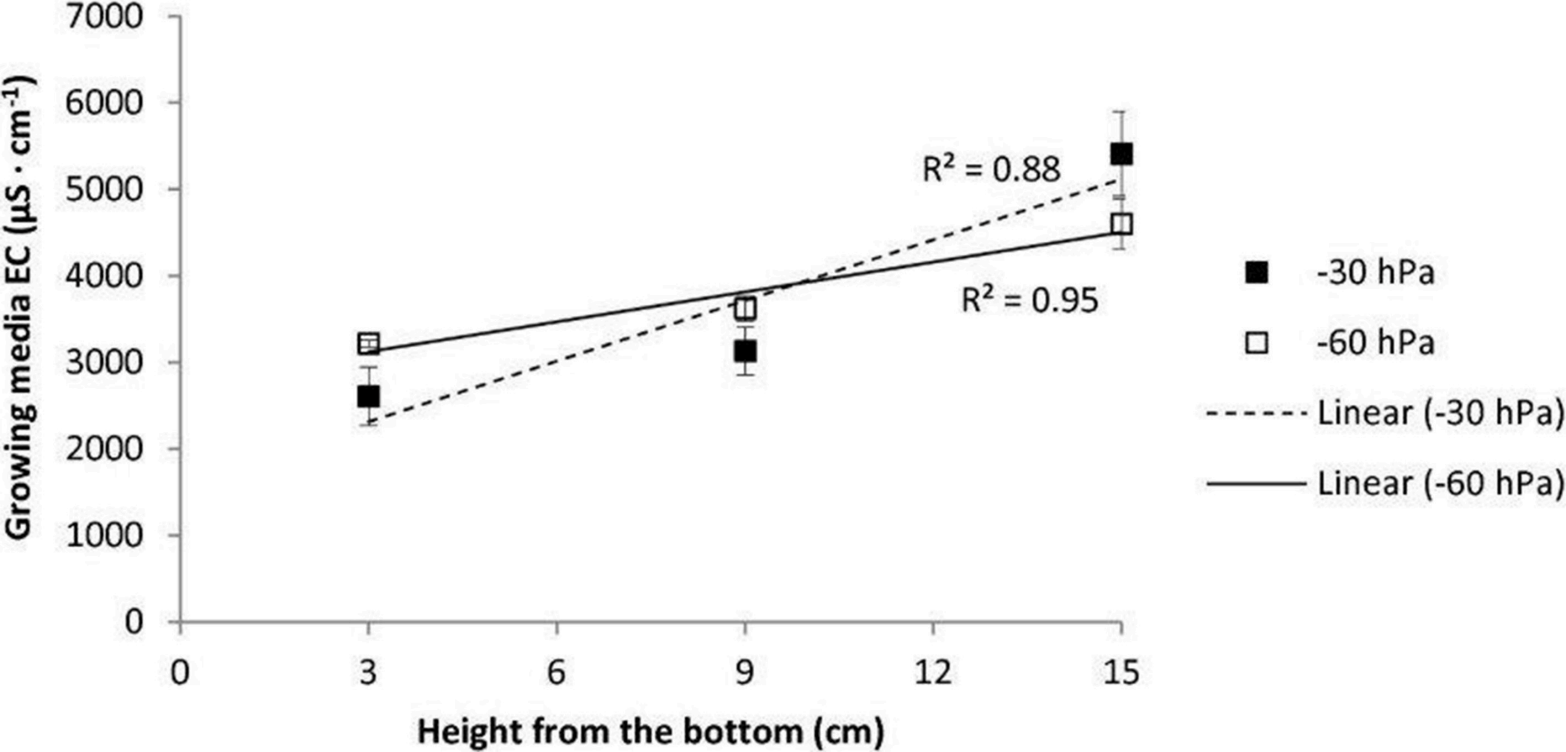
**Electrical conductivity (EC, 1:1.5 dilution method) at three layers of the vertical profile in subirrigated soilless substrates with constant −30 or −60 hPa matric potential irrigation set-point (vertical bars indicate ±S.E. of means)**.

Growing media of all treatments showed the salt stratification typical of subirrigated substrates. EC rose from the bottom to the top layer, with a higher increase in the trend when a −30-hPa matric potential was maintained in the growing media (Figure 1). The lower moisture content of substrates subjected to the lowest matric potential limited the upward salt flux, with a consequent higher EC in the bottom and middle layers of the substrate (3.2 and 3.6 vs. 2.6 and 3.1 dS/m, respectively, in the bottom and middle layers of −60 and −30 hPa substrates) and lower EC in the top layer (4.6 vs. 5.4, respectively, for −60 and −30 hPa substrates), resulting in a reduced EC gradient over the substrate profile.

### Plant growth and yield

Water potential in the growing media affected tomato plant growth without significant interaction between the cultivar and water stress (Table 2). When comparing −30 with −60 hPa, a mean reduction of 18% for leaf area and 19% for SLA were observed, whereas plant height and total dry weight were unaffected. A further reduction in matric potential from −60 to −90 hPa in “Kabiria” had no effects on plant growth (Table 2). “Kabiria” showed, on average, higher plant height but lower leaf area and SLA than “Diana.” For both cultivars, the lowering of the water potential in the growing media from −30 to −60 hPa led to a reduction in total yield (−10%, on average) and in mean fruit size (−13%, on average), with no interaction between cultivar and water stress; in the case of “Kabiria,” where also a potential of −90 hPa was tested, the reduction of those two parameters followed a linear trend with the lowering of water availability in the growing media. “Diana” showed, on average, the highest total yield (3.1 vs. 2.7 kg·plant^−1^, on average) and fruit mean weight (106 vs. 60 g) (Table 2).

**Table 2:** **Effect of cultivar (Diana—D and Kabiria—K) and growing-media matric potential (P) on plant height, leaf area, specific leaf area (SLA), total dry weight, total and unmarketable yield, and mean fruit weight of subirrigated soilless tomato under controlled water stress conditions**.

**Cultivar-Water potential (hPa)**	**Plant height (cm)**	**Leaf area (cm^2^)**	**SLA (cm^2^·g^−1^)**	**Plant dry weight (g·plant^−1^)**	**Total yield (g·plant^−1^)**	**Unmarketable yield (g·plant^−1^)**	**Mean fruit weight (g)**
D-P30	164	10,989	67.1	164	3293	187	112
D-P60	161	8811	49.9	180	2997	349	99
K-P30	204	7271	41.9	174	2851	145	64
K-P60	204	6078	36.6	166	2532	108	55
K-P90	199	6137	38.0	163	2050	82	49
*Significance of contrasts*^a^**							
30 vs. 60 hPa	ns	^*^	^*^	ns	^**^	^**^	^**^
D vs. K	^***^	^**^	^**^	ns	^**^	^***^	^***^
(D vs. K)^*^(30 vs. 60 hPa)	ns	ns	ns	ns	ns	^***^	ns
P (K) linear	ns	ns	ns	ns	^***^	^*^	^**^
P (K) quadratic	ns	ns	ns	ns	ns	ns	ns

a F significance: ns, ^***^, ^**^, and ^*^, respectively, non-significant, P ≤ 0.001, P ≤ 0.01, and P ≤ 0.05; fw, fresh weight.

Different water potential conditions in the growing media had opposite effects on the incidence of unmarketable yield for the two cultivars: while for “Diana” the unmarketable percentage rose from 5.7 to 11.6% of total yield, respectively, at −30 and −60 hPa, and for “Kabiria” the percentage decreased with the lowering of substrate water potential, following a linear trend (Table 2).

In both cultivars, the number of fruits per plant was not affected by water stress, with a mean value of 30 and 44, respectively, for “Diana” and “Kabiria” (data not reported).

### Fruit quality

In the second cluster, for both cultivars, the lowering of growing media water potential improved fruit quality in terms of TSS and dry matter, with a significant increase in those two parameters (5 and 12.5% on average, respectively) with no interaction between cultivar and water stress (Table 3). In the case of “Kabiria,” both TSS and dry matter increased linearly with decreasing growing media water potential. Both glucose and fructose concentrations increased linearly in “Kabiria” fruits with decreasing substrate matric potential, whereas the concentration was not affected by water stress in “Diana.” The inorganic ion concentration in the fruit (K^+^, Mg^2+^, and Ca^2+^) was not significantly affected by the matric potential irrigation set point, although a certain trend of increase in K^+^ was observed (Table 3).

**Table 3:** **Effect of cultivar (Diana—D and Kabiria—K) and growing-media matric potential (P) on total soluble solids (TSS), fruit dry matter, glucose, fructose, K, Mg, and Ca fruit concentration in the second and seventh cluster of subirrigated soilless tomato under controlled water stress conditions**.

**Cultivar-Water potential (hPa)**	**TSS (°Brix)**	**Dry matter (g·100 g^−1^ fw)**	**Glucose (mg·100 mL^−1^ fruit juice)**	**Fructose (mg·100 mL^−1^ fruit juice)**	**K^+^ (mg·kg^−1^ fw)**	**Mg^2+^ (mg·kg^−1^ fw)**	**Ca^2+^ (mg·kg^−1^ fw)**
**SECOND CLUSTER**							
D-P30	5.0	5.8	0.80	1.57	1823	44	52
D-P60	5.4	6.7	0.80	1.50	2098	49	45
K-P30	6.7	7.4	1.10	1.03	1387	35	46
K-P60	6.9	8.1	1.20	1.40	1426	31	31
K-P90	7.2	8.7	1.40	1.65	1681	35	31
*Significance of contrasts^a^*							
30 vs. 60 hPa	^*^	^**^	ns	ns	ns	ns	ns
D vs. K	^***^	^***^	^***^	^**^	^*^	^*^	ns
(D vs. K)^*^(30 vs. 60 hPa)	ns	ns	ns	^*^	ns	ns	ns
P (K) linear	^*^	^**^	^**^	^***^	ns	ns	ns
P (K) quadratic	ns	ns	ns	ns	ns	ns	ns
**SEVENTH CLUSTER**							
D-P30	6.1	7.8	–	–	2589	58	58
D-P60	6.3	7.9	–	–	2463	60	51
K-P30	6.9	8.3	–	–	2321	47	39
K-P60	7.5	9.3	–	–	2700	56	41
K-P90	8.3	10.5	–	–	2772	56	42
*Significance of contrasts^a^*			–	–			
30 vs. 60 hPa	^*^	^**^	–	–	ns	^*^	ns
D vs. K	^***^	^***^	–	–	ns	^**^	^***^
(D vs. K)^*^(30 vs. 60 hPa)	^*^	^*^	–	–	ns	ns	ns
P (K) linear	^***^	^***^	–	–	ns	ns	ns
P (K) quadratic	ns	ns	–	–	ns	ns	ns

a F significance: ns, ^***^, ^**^, and ^*^, respectively, non-significant, P ≤ 0.001, P ≤ 0.01 and P ≤ 0.05; fw, fresh weight.

In the seventh cluster, the two cultivars reacted differently to water stress application in terms of TSS and dry matter. In “Diana” only a slight increase was observed from −30 to −60 hPa (3.3 and 1.3%, respectively, for TSS and dry matter), whereas in “Kabiria,” the increase was more pronounced (8.7 and 12.0%, respectively, for TSS and dry matter), and further reduction in matric potential from −60 to −90 hPa in “Kabiria” confirmed the linear increase for both the parameters. The fruit concentration of Mg^2+^ tended to slightly increase in both cultivars under water stress conditions (Table 3).

### Plant–water relations

No interaction was observed between cultivar and water stress with regard to plant–water relations. Regardless of the cultivar, water stress decreased Ψ_i_ and Ψ_x_, with more remarkable differences in measurements taken at noon because of the greater influence of environmental conditions (Table 4). In the case of “Kabiria,” Ψ_i_ and Ψ_x_ decreased following a linear trend with lowering of growing media water potential from −30 to −90 hPa. Plants of both cultivars showed an osmotic adjustment with a 9% decrease, on average, in Ψ_o_-values from −30 to −60 hPa. No differences in Ψ_t_ were observed in plants (Table 4).

**Table 4:** **Effect of cultivar (Diana—D and Kabiria—K) and growing-media matric potential (P) on water (Ψ_**i**_), osmotic (Ψ_**o**_), and turgor (Ψ_**t**_) pre-dawn potential and noon water potential on covered leaf (Ψ_**x**_) of subirrigated soilless tomato under controlled water stress conditions**.

**Cultivar-Water potential (hPa)**	**Ψ_i_**	**Ψ_o_**	**Ψ_t_**	**Ψ_x_**
	**(MPa)**			
D-P30	−0.263	−0.953	0.690	−0.280
D-P60	−0.353	−1.023	0.669	−0.483
K-P30	−0.247	−0.922	0.675	−0.313
K-P60	−0.327	−1.021	0.694	−0.457
K-P90	−0.510	−1.093	0.583	−0.733
*Significance of contrasts^a^*				
30 vs. 60 hPa	^*^	^**^	ns	^**^
D vs. K	ns	ns	ns	ns
(D vs. K)^*^(30 vs. 60 hPa)	ns	ns	ns	ns
T (K) linear	^**^	^**^	ns	^***^
T (K) quadratic	ns	ns	ns	ns

a F significance: ns, ^***^, ^**^, and ^*^, respectively, non-significant, P ≤ 0.001, P ≤ 0.01 and P ≤ 0.05.

### Water consumption and water-use efficiency

Water consumption was similar for both cultivars and decreased with decreasing growing media matric potential (104 and 89.5 L·plant^−1^, on average, at −30 and −60 hPa, respectively—Table 5). The water consumption reduction in “Kabiria” followed a linear trend with the lowering of the growing media water potential (Table 5). WUE was unaffected by water potential but was 16% higher in “Diana.”

**Table 5:** **Effect of cultivar (Diana—D and Kabiria—K) and growing-media matric potential (P) on total water consumption and water-use efficiency (WUE) of subirrigated soilless tomato under controlled water stress conditions**.

**Cultivar-Water potential (hPa)**	**Water consumption (L·plant^−1^)**	**WUE (g·L^−1^)**
D-P30	107	31
D-P60	89	34
K-P30	101	28
K-P60	90	28
K-P90	73	28
*Significance of contrasts^a^*		
30 vs. 60 hPa	^***^	ns
D vs. K	Ns	^**^
(D vs. K)^*^(30 vs. 60 hPa)	Ns	ns
P (K) linear	^***^	ns
P (K) quadratic	Ns	ns

a F significance: ns, ^***^, and ^**^, respectively, non-significant, P ≤ 0.001, and P ≤ 0.01.

## Discussion

In this study, we combined the tools for efficient water use and satisfactory crop performance in soilless greenhouse tomato cultivation. With this aim, a closed-cycle through bench subirrigation technique and on-demand sensor-based irrigation was used.

Subirrigation is an easy-management closed-cycle technique, which is suitable to be used also in low-tech greenhouse industry (Bouchaaba et al., 2015). As a closed-cycle system, the technique itself is an effective way of reducing water usage and nutrient runoff. However, proper irrigation management is required for the success of such a technique, and identification of proper irrigation set points is crucial for optimal crop performance, both in terms of yield and quality, and optimal use of water resources. In the present study, we demonstrated that sensor-based irrigation management can help implement a closed-cycle cultivation of soilless tomato using subirrigation. In addition, precise control of substrate water status may offer the possibility to steer crop response by enhancing two different components of crop performance, namely yield and fruit quality.

Maintaining the optimal water status of soilless growing media has been recognized as being critical, both because of its limited water-holding capacity and the difficulty in judging accurately when the plants require water (Fonteno et al., 1981; Karlovich and Fonteno, 1986). Substrate matric potential measurement and control through a tensiometer can be used with this aim, with the main advantage of directly determining whether water in the soil is available to the plants (van Iersel et al., 2013). In the present study, we used a tensiometer to completely automate the irrigation management of the crop and to impose controlled water stress corresponding to the different irrigation set points based on substrate matric potential. Although a generally accepted theory establishes the general matric potential value limits for EAW in soilless substrates, thus providing guidance for determining optimal irrigation set points, there are only a few specific studies that relate the cultivation technique and the response of the different crops and varieties to different matric potential values. The results of the present research outline the general negative effects of matric potential decrease on growth and total yield of subirrigated tomato, showing that an irrigation set point exceeding the conventional limit of −50 hPa leads to a reduction in growth and total yield (Table 2).

The differences in height, leaf area, and SLA between the two tested varieties are due to their different characters: “Diana” being characterized by lower plant height and higher leaf area than “Kabiria.” Although “Diana” presented higher leaf area than “Kabiria,” water use for the two cultivars was similar (Table 5). Our data seem to confirm that leaf area value is not an exhaustive parameter to explain differences in water use by plants of different cultivars, but it should be related to the plant's character. In a previous study, Santamaria et al. (2004) found that “Diana” and “Naomi,” a cherry tomato variety, presented similar leaf area, but “Diana” consumed 8% less water than “Naomi.” In the present study, we found the water consumption per unit of leaf area of 9.8 and 14.3 mL·cm^−2^, respectively, for “Diana” and “Kabiria.” The higher leaf area distributed in a shorter plant height may have resulted in higher shading of “Diana” leaves compared with “Kabiria,” affecting the water consumption rate of the two varieties.

Although for most of the tested parameters the interaction between cultivar and water stress level was not significant, according to our findings, the specific response of the cultivar to controlled water stress, in terms of important crop-performance indicators, should be taken into account. In fact, while the unmarketable yield in “Diana” increased when water stress was imposed (Table 2) mainly due to blossom-end rot, an opposite effect was observed in “Kabiria,” where marketable yield loss linearly decreased by 1.05 g·plant^−1^ per unit of substrate water potential (in the tested range from −30 to −90 hPa) mainly due to a reduced occurrence of fruit cracking, the latter variety being characterized by small-sized fruits and probably higher resistance to water stress. The reduced tolerance to water stress in “Diana,” in terms of incidence of unmarketable fraction of the yield, confirmed the higher susceptibility to water stress of cultivars with large or intermediate fruits. Conversely, “Kabiria” showed higher adaptation to water stress conditions, typical of small-sized fruit genotypes (Santamaria et al., 2004; Serio et al., 2007).

Previous studies on drip-irrigated tomatoes cultivated in soilless peat–perlite mix-based (70:30, v:v) substrates showed a 6% yield reduction when using a −100-hPa compared to a −50-hPa irrigation trigger set-point (Xu et al., 1995), whereas no differences were observed when comparing −45 and −65 hPa matric potentials (Norrie et al., 1995). Sarkar et al. (2008a) found a progressive decrease in yield and growth parameters in tomato plants cultivated in an open-cycle drip-irrigated tomato on coir dust substrate. Beside the important effects on crop performance related to the adoption of proper matric potential irrigation set points, irrigation management using tensiometers reduced leaching and the amount of greenhouse effluents in a soilless tomato cultivation by 262 kg N·ha^−1^ when compared with the use of timer-based irrigation adjusted to solar radiation (Lemay et al., 2012).

The tensiometer has been previously used for the automated irrigation management of subirrigated containerized vegetables in some studies (Montesano et al., 2010; Bouchaaba et al., 2015), although only in rare cases specific comparisons between different water potential set points have been reported (Montesano et al., 2005a,b). In those studies, when comparing an irrigation set point of −40 hPa relative to −80 hPa, subirrigated tomato “Kabiria” grown on a perlite–peat mix similar to that used in the present research showed a reduction of 26% in yield and 16% in mean fruit weight, an increased dry matter percentage and TSS of fruits with the lowest matric potential, whereas WUE was not affected. Morever, in the present research, WUE was not affected by the matric potential control. In fact, both terms of WUE ratio (yield and water use) were similarly reduced when water stress (−60 hPa) was applied, without significant differences among varieties (Table 5).

Wang et al. (2007) claimed an increased tomato plant WUE and irrigation system WUE as soil matric potential decreased, with reduced irrigation amounts and no significant differences in tomato yield; however, the study was conducted on soil and a wide range of matric potential was tested (−10 to −50 kPa). WUE can be improved by modifying both terms of the ratio (De Pascale et al., 2011), but according to our findings, acting on matric potential control to impose controlled water stress is not a suitable approach for improving this parameter in subirrigated soilless tomato, thereby confirming that in soilless conditions, due to the low water-holding capacity of growing media, even slight decreases in soil matric potential could lead to severe effects on plant physiology and crop performance.

Tomato fruit quality, which is controlled by the interaction of genetic, environmental, and cultural factors, has become a major commercial concern in the highly competitive fresh fruit market, with increasing attention to setting up growing conditions and specific interventions capable of enhancing quality. In particular, there is an ever-increasing demand for high soluble solid content (Sarkar et al., 2008b). In the present study, a significant effect of controlled water stress on plants subjected to low water potentials lies in the improvement in fruit quality traits of commercial importance, namely TSS and dry matter content (Table 3). The two tested cultivars reacted differently in terms of benefit of water stress on fruit quality, as confirmed in particular by the quality traits analysis performed on the seventh cluster, where only slight quality improvements were observed in “Diana” (Table 3). A significant increase in TSS and fruit dry matter content was always observed in “Kabiria.” In this cultivar, as a result of matric potential decrease from −30 to −60 hPa, the fructose/glucose ratio increased (from 0.94 to 1.17, respectively), whereas further decrease in substrate water potential (−90 hPa) did not result in further increases of this parameter. However, considering the sweetness index [sweetness index (SI) calculated by multiplying the sweetness coefficient for each individual sugar (glucose = 1, fructose = 2.3) as described by Keutgen and Pawelzik (2007)], we found that SI increased by 26 and 49%, respectively, at −60 and −90 hPa, when compared to −30 hPa. This indicates that the effect of controlled water stress on tomato quality amelioration may involve effects on the ratio between the two principle reducing sugars present in fruits. Moreover, the higher benefit for small or medium-sized fruit tomato types seems to be confirmed in terms of quality amelioration as a response to controlled water stress (Elia et al., 2001; Di Gioia et al., 2013). For large fruit varieties, the commercial maturity of fruits corresponds to the “breaker” color stage, whereas smaller varieties are generally harvested when fruits are red-ripened. The fact that fruits of small-sized varieties are allowed to ripen on the plant before harvest may help maximize the effects of controlled water stress in terms of TSS and dry matter increase.

A common practice for fruit quality improvement in tomato is to apply controlled stress to plants by using saline water, high concentrated NS, or slight water stress in order to obtain fruits with higher dry matter and TSS concentration, although such practices generally lead to yield loss (Dorais et al., 2000; Guichard et al., 2001). In subirrigation conditions, since no leaching occurs in containers, salt accumulation, in particular at the substrate surface, is a major drawback (Elia et al., 2003; Santamaria et al., 2003; Rouphael and Colla, 2005). Therefore, many authors suggest reducing fertilizer NS concentration in subirrigation as compared to traditional drip irrigation systems (Cox, 2001; Mak and Yeh, 2001; Yeh et al., 2004; Montesano et al., 2010). However, an important effect of matric potential irrigation set point on substrate EC and salt stratification was found in the present study. In particular, lower water potential conditions impaired the typical stratification of unabsorbed salts in the substrate, leading to higher EC levels at the bottom and middle of the substrate profile (Figure 1). The accumulation of salts in the top surface layer of the substrate, and the consequent lower accumulation at the bottom where most of the roots are present, has often been reported as a natural counterbalance to the absence of leaching that occurs in subirrigation (Morvant et al., 1997; Bouchaaba et al., 2015). Therefore, it is important to take into account the possible effects of irrigation management conditions on the occurrence of such a phenomenon, in particular when saline water or unbalanced fertilizer solutions are used in subirrigation, with potential implications on salt stress for plants. Given such considerations, the common practice of using highly concentrated NS, as well as using NS containing NaCl, to obtain high-quality tomato fruits does not seem to lack possible negative implications when subirrigation techniques are adopted (Incrocci et al., 2006). However, according to the present study findings, it is feasible to act on matric potential irrigation set points to control the response of plants in terms of fruit quality parameters.

At the physiological level, as a consequence of the moisture decrease in the root zone, plants of both cultivars showed an osmotic adjustment (lowering of leaf osmotic potential—Table 4). Osmotic adjustment is an adaptive physiological mechanism involved in stress tolerance to drought and salinity, which permits the maintenance of turgor and cell functions under conditions of water deficit. It is reported that under salinity stress, this process is achieved mainly by uptake and accumulation of inorganic ions, whereas under drought stress, it is achieved by synthesis and accumulation of organic compatible solutes. However, it has been reported that drought tolerance in tomato varieties is not always correlated to tissue osmotic potentials, implying that osmotic adjustment is not the only process influencing tolerance (Alian et al., 2000).

A more pronounced tendency to overcome water stress by osmotic adjustment mechanisms was observed in “Kabiria,” which showed a significant increase in glucose (5 μg·100 mL^−1^ fruit juice per unit of substrate water potential in the tested range from −30 to −90 hPa) and fructose (10.3 μg·100 mL^−1^ of fruit juice per unit of substrate water potential in the tested range) fruit concentrations under water stress conditions, whereas in “Diana,” no significant changes in the concentration of those two sugars were observed as a result of water stress (Table 3). The role of accumulation of solutes in the cell (mostly compatible solutes, sugars or amino-acids in the cytoplasm, and inorganic solutes in the vacuole) for the osmotic adjustment has been demonstrated (Kramer and Boyer, 1995). However, while Veit-Kohler et al. (1999) claimed that a reduction in water supply led to an increase in sugars and titratable acids in tomato fruits, Nahar and Gretzmacher (2002) found similar responses only under extreme water deficit conditions, and Plaut et al. (2004) concluded that solutes contributing to the osmotic adjustment of fruits and leaves were only slightly affected by water stress.

## Conclusions

Substrate matric potential control using a tensiometer can be used for automatic irrigation in soilless subirrigation systems. An irrigation set point exceeding the conventional limit of easily available water for soilless substrates leads to reduction of growth and total yield of subirrigated soilless tomato. Precise control of substrate water status may offer the possibility to steer crop response by enhancing different crop-performance components, namely yield and fruit quality. Small-sized fruit varieties benefit more from controlled water stress in terms of reduced unmarketable yield loss and fruit quality traits improvement.

### Conflict of interest statement

The authors declare that the research was conducted in the absence of any commercial or financial relationships that could be construed as a potential conflict of interest.
